# Operative management of immune checkpoint colitis following in-transit melanoma: Case report

**DOI:** 10.3389/fonc.2023.1120808

**Published:** 2023-04-20

**Authors:** Betzaira G. Childers, Eileen Donovan, Winifred M. Lo, Lauren M. Janowak, Jeffrey Sussman, Christopher F. Janowak

**Affiliations:** ^1^ College of Medicine, University of Cincinnati, Cincinnati, OH, United States; ^2^ Department of Surgery, University of Pittsburgh Medical Center, Pittsburgh, PA, United States; ^3^ College of Nursing, University of Cincinnati, Cincinnati, OH, United States

**Keywords:** immune-checkpoint-inhibitor, colitis, melanoma, surgery, complications, hidradenitis

## Abstract

Immune checkpoint inhibitors are increasingly used as powerful anti-neoplastic therapies in the setting of melanoma. Colitis is a known complication of immune checkpoint inhibitors that if often medically managed. We present a patient with stage IV melanoma with demonstrated in-transit disease undergoing immune checkpoint inhibitor therapy. The patient subsequently developed recalcitrant severe colitis that necessitated operative intervention and bowel resection. The association of immune check point inhibitors and immune related adverse effects are discussed as well as treatments of advanced colitis, including the possibility of surgical management in the setting of severe colitis with complications.

## Introduction

Harnessing the immune system’s ability to fight infection is a gateway to controlling and overcoming malignancy. Immune checkpoint inhibitors (ICI) are a powerful class of medications that have made use of this potential for melanoma since receiving Federal Drug Administration approval in 2015. Since then, several immune checkpoints have been approved for treatments including advanced and refractory disease. Given their systemic distribution, ICI can precipitate widespread immune related adverse events (iRAEs) including rashes, hypothyroidism, transaminitis, blindness, pancreatitis, diarrhea, & colitis. In a retrospective study of 70 patients receiving Nivolumab monotherapy for non-small cell lung cancer, 40% of patients developed iRAEs ranging from skin reactions to pneumonitis (1). Similarly, a retrospective review of 148 patients who received Nivolumab treatment for melanoma as a part of phase I clinical trials found that 68.2% of patients experienced an iRAE (2). While low-grade side effects can be monitored or treated medically, more severe iRAEs such as colitis have the potential for rapid progression that may necessitate surgical intervention. In patients treated with anti PD-1/PD-L1 therapy, the reported incidence of colitis overall is 1.3-1.6%, while high-grade colitis is quite rare, occurring in approximately 0.9% of patients (3, 4). However, the relative risk of high-grade ICI colitis is higher in dual agent ICI therapy than single agent ICI (5). Overall, data is evolving regarding the optimal strategies for treating for these complex patients. Here, we present a rare case of severe colitis secondary to treatment with immunotherapy for melanoma that required surgical intervention.

## Case

**Figure 1:**
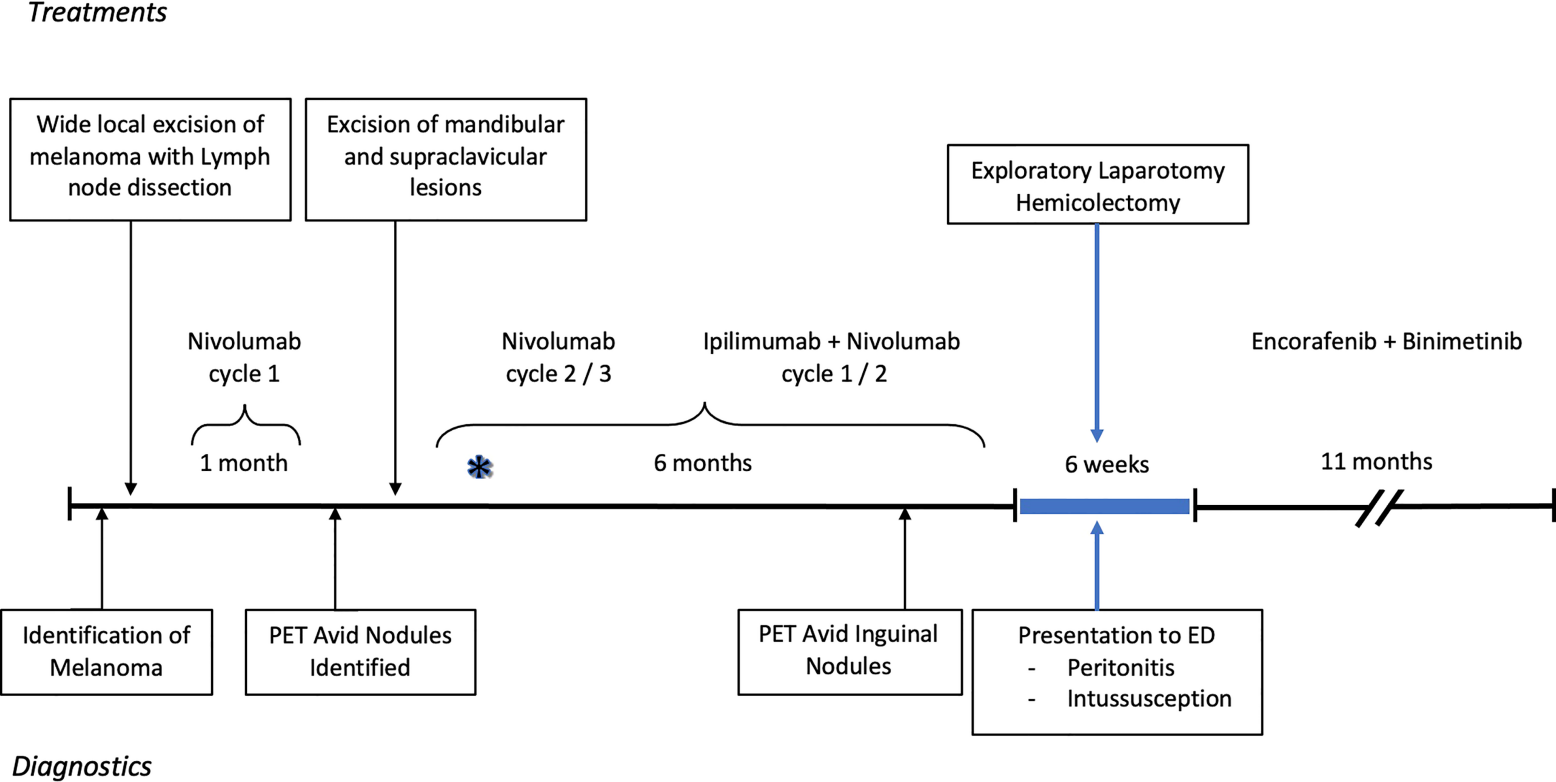
Timeline of diagnostics and treatment of melanoma and subsequent severe colitis resulting in surgery. The asterisk reflects when the patient first noticed colitis symptoms. The PET, positron emission tomography; ED, Emergency department.

Our patient is a 37-year-old male with a history of hidradenitis and initially found to have stage III melanoma that was diagnosed *via* punch biopsy of a suspicious mid-upper back mole. The patient has a father who had a skin cancer of unknown type removed from his nose, but no other familial oncologic history. The patient’s lesion was incidentally noticed in a tattooed area of skin. The biopsy revealed a 5.7 mm thick, non-ulcerated, BRAF positive, malignant melanoma with >20 mitoses per high powered field. Pathology following wide local excision with axillary lymph node biopsy revealed a melanoma with a final depth of 7 mm and 1 of 4 positive lymph nodes consistent with stage III disease. He was started on Nivolumab 100 mg and enrolled in the Elios Vaccine trial. During the first month of therapy the patient was noted to have multiple skin nodules in addition to hypermetabolic regions on positron emission tomography (PET) scan in a right supraclavicular node, bilateral axillae, and perineum. Changes seen in the axillae and perineum were found to be consistent with the patient’s cystic acne and hidradenitis. Due to concern for metastases, a right mandibular lesion and supraclavicular node were excised. The mandibular lesion was found to be benign, but the supraclavicular node was positive for melanoma. He was then upstaged to stage IV disease and therapy was escalated to Ipilimumab plus Nivolumab per the NCCN guidelines (6). He completed 3 cycles of Nivolumab and two cycles of Ipilimumab plus Nivolumab with side effects including palpitations, rash, nausea, diarrhea, and recurrent grade 3 colitis over the course of 8 months. As treatment for the side effects, he received escalating doses of prednisone up to 100 mg daily. Repeat PET scan 6 months after diagnosis demonstrated hypermetabolic foci of uptake in the inguinal and pubic regions; however, this was correlated to areas of hidradenitis-related inflammation (**Figure 1**).

**Figure 2:**
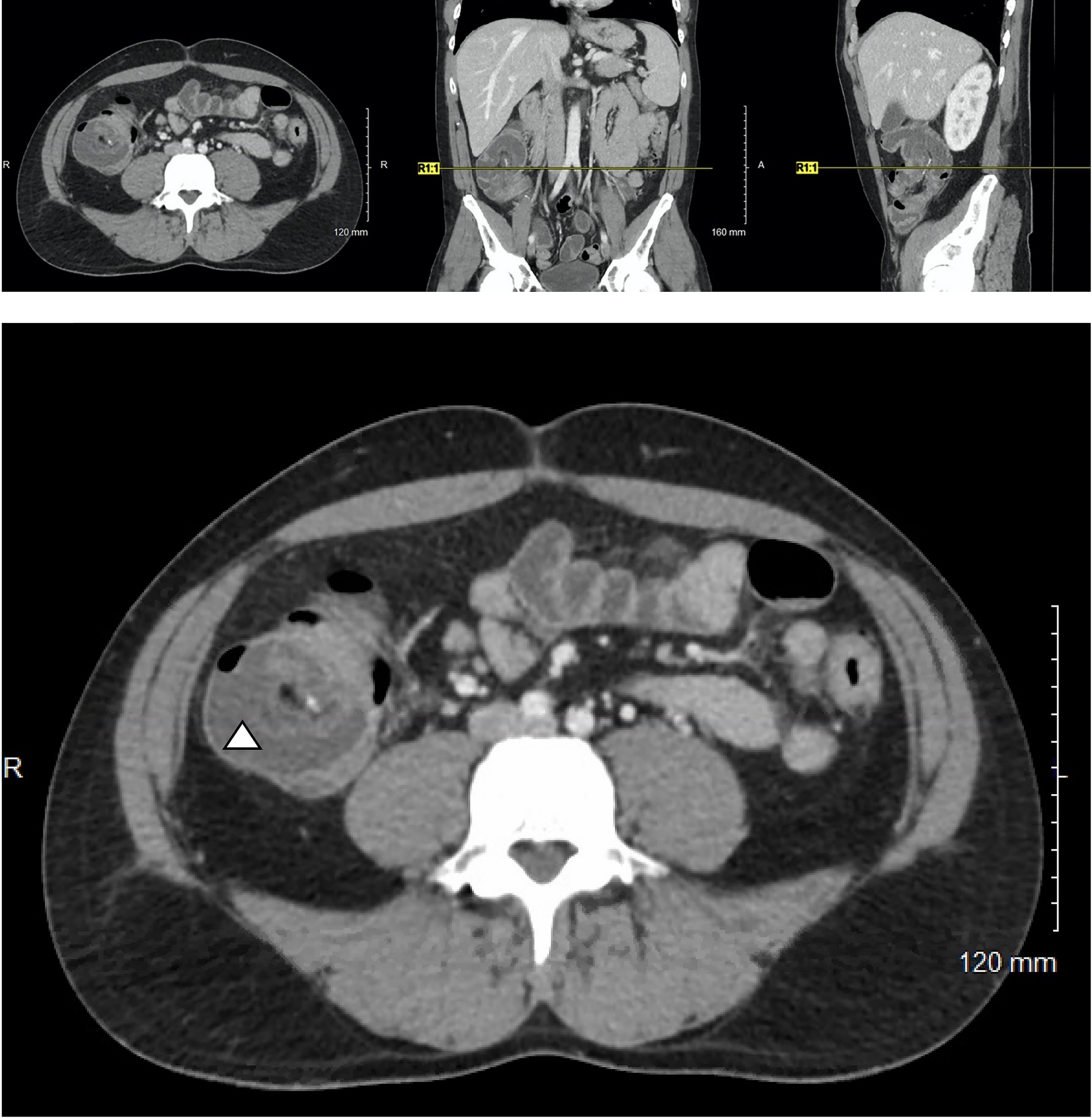
Cross-sectional imaging of a 37-year-old M with Stage IV in-transit melanoma receiving immune checkpoint inhibitor therapy who presents with severe abdominal pain. A characteristic target-sign for intussusception is appreciated in cecum (triangle) and can be seen on the axial, coronal, and sagittal views.

**Figure 3:**
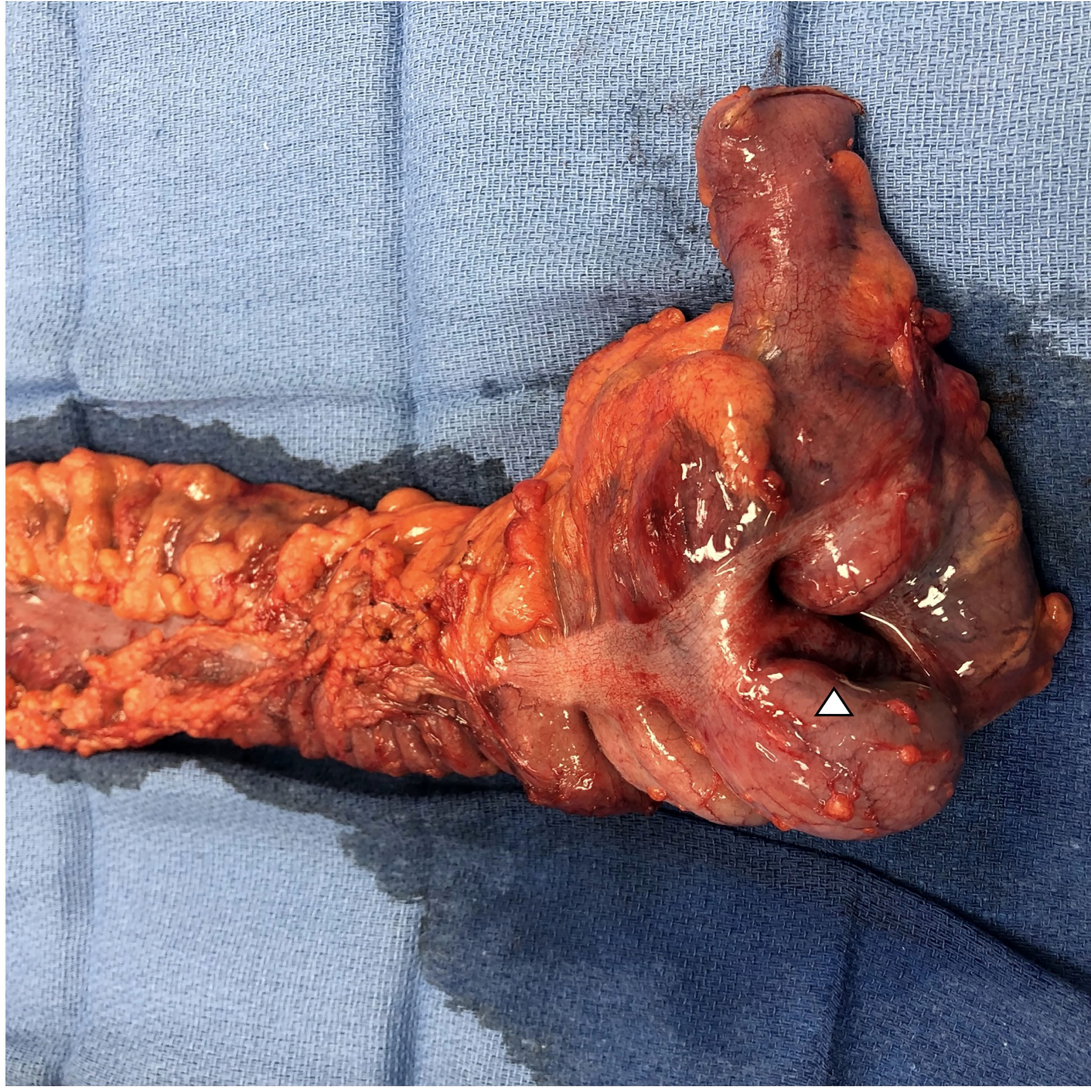
Photo of surgical resection specimen with terminal ileum, ileocecal valve, cecum, and ascending colon. The triangle denotes the location of the intussusception that appears to originate at the level of the coalescence of the tinea at the base of the cecum. Final pathology demonstrated advanced colitis and cecal ischemia.

Soon thereafter, he presented to the emergency department with 24 hours of fatigue, weakness, peritoneal abdominal pain, and diarrhea that progressed to bright red blood per rectum. Cross-sectional imaging at the time demonstrated ileocecal intussusception and raised concerns for a cecal mass (**Figure 2**) suggesting neoplastic disease. Due to peritonitis, he was taken to the operating room and underwent an exploratory laparotomy. Intraoperative findings identified significant cecal induration, marked inflammation, patchy areas of ischemia, and the appearance of cecal intussusception. Specifically, the cecum appeared to invaginate from the coalescence of the tinea (where the appendiceal orifice would have been; however, the patient had a laparoscopic appendectomy years prior) and had intussuscepted antegrade along the ascending colon (**Figure 3**). There were signs of colonic ischemia, but no gangrene or perforation, and the affected area was resected with right hemicolectomy. Intestinal continuity was restored with an ileocolonic anastomosis. The postoperative course was complicated by a minor wound seroma and colitis with diarrhea that resolved with conservative management. The final surgical pathology was consistent with localized colonic ischemia and necrosis due to intussusception, with surrounding colitis. No evidence of malignancy was identified, and 30 reactive lymph nodes were recovered without evidence of metastasis. Notably, the lead point for the intussusception appeared to be at the site of the appendiceal stump from his prior appendectomy. At the time of manuscript preparation, the patient is doing well having completed encorafenib and binimetinib treatments and has had no further evidence of disease recurrence.

The patient has graciously given his permission for the use of his story to help enrich the understanding of disease. Informed consent obtained and CARE checklist completed. From the patient’s perspective, the course of his melanoma disease was rapid, raising concerns from his treatment team early on as to whether his initial presentation represented Stage III vs. Stage IV disease. He also recalls that the timing of the colitis was “nearly instantaneous” with the initiation of combination Ipilimumab and Nivolumab therapy (indicated on **Figure 1** with an asterisk). Symptom management with steroids was ineffective and the colitis continued to accelerate until it became a surgical emergency and ultimately one of the scariest chapters of his life. Coincidentally, the immunotherapy also exacerbated his hidradenitis disease, adding to the complexity of maintenance type therapies during this period. Through everything he has remained optimistic and recovered remarkably well.

## Discussion

We present a case of melanoma treatment with ICIs that is complicated by a severe manifestation of colitis. While ICI colitis is not specific to melanoma, much of the growing body of evidence is based on melanoma due to the timing of ICIs as leading agents in the treatment of advanced stage melanoma. However, their use in other cancer types is growing and the incidence of cancer specific colitis remains to be seen. Overall, the incidence of colitis in patients undergoing treatment with immunotherapy is relatively uncommon, with a highest reported incidence of 13.6%, but the diagnosis requires prompt intervention and treatment due to the associated risk of significant morbidity and mortality (4, 7). Our understanding for the severity and incidence of iREAs is evolving and currently in-completely understood. The use of ICIs across various cancer types is expanding and emphasizes the importance of vigilant surveillance as criteria to predict what patients will fail conservative treatment is poorly understood. Tandon et al, identified five aCTLA4 colitis deaths though their specific clinical course and or candidacy for operative intervention is unclear (3). Additional work is needed to identify patient populations who will fail conservative management and will benefit from escalation of care to include operative intervention. Even after a period of cessation, iRAEs can reoccur in up to one third of patients after resumption of either an aCTLA4 or aPDL1. Less severe symptoms were associated with aPDL1 while aCTLA4 frequently required corticosteroids and anti-tumor necrosis factor alpha agents. The rate of diarrhea colitis recurrence appears to be higher in patients undergoing aPDL1 therapy (8). In addition, in randomized control trials the relative risk of high-grade ICI colitis is higher at 1.33 in dual therapy Ipilimumab + Nivolumab vs Ipilimumab alone (5). Continued work is necessary to better delineate the risks associated with single, combination, and repeated treatment regimens. Notably, iRAEs and specifically gastrointestinal related adverse events of any grade are associated with a statistically significant survival benefit (p<0.01). This is despite the use of immunosuppressive medication in severe cases (9). Unfortunately, even less is known about the immune modifying effects of surgery for those with severe, life-threatening colitis as is the case with our patient. For example, the typically immune suppressive effect of surgery on cancer during the perioperative period has been implicated in tumor escape and expansion (10, 11). Perioperative dosing of cytokines, toll-like receptor agonists, anti-catecholamines, anti-prostaglandins, and immune checkpoint medications have shown the potential to extend the progression free survival in animal models (9).

Beyond immunotherapy mediated colitis, operative intervention for any colitis events remains uncommon but is associated with significant morbidity. In one study, surgical resection was required in approximately 17% of all patients presenting with ischemic colitis (Yadav et al) (12). In a review of 4548 patients undergoing emergent colectomy for ischemic colitis of any etiology, 30-day post-operative mortality was found to be greater than 25% (Tseng et al) (13). In this cohort, disseminated cancer (p < 0.001) and chronic steroid use (p < 0.001) were both associated with increased mortality on univariate analysis. As the use of ICIs increases the development and use of escalating treatment guidelines for immune mediated colitis (14) may offer insights towards the prevention of severe colitis. However, at this point a patient undergoing immunotherapy for cancer who develops colitis necessitating operative intervention should be considered high-risk for poor post-operative outcomes. Colitis surveillance and mitigation may help avoid operative intervention and or guide timing of intervention to maximize patient outcomes.

## Conclusion

Indications for ICI use in various malignancies are increasing. While low-grade side effects can be monitored and symptomatically treated, severe iRAEs can progress quickly and lead to complications that may require surgical intervention. Refractory colitis may portend surgical emergencies and should be evaluated with a high suspicion for operative intervention. More work is needed to understand the incidence of colitis progression in the setting of single and multiple ICI combinations for malignancy.

## Data availability statement

The original contributions presented in the study are included in the article/supplementary material. Further inquiries can be directed to the corresponding author.

## Ethics statement

Written informed consent was obtained from the participant/patient(s) for the publication of this case report.

## Author contributions

WL, JS, and CJ contributed to the design of the study. BC, ED, LJ, and CJ contributed to the literature search and acquisition of data. BC, ED, LJ, and CJ drafted the manuscript. All authors contributed to the article and approved the submitted version.
